# “REOFUT” as an Observation Tool for Tactical Analysis on Offensive Performance in Soccer: Mixed Method Perspective

**DOI:** 10.3389/fpsyg.2019.01476

**Published:** 2019-06-28

**Authors:** Rafael Aranda, Joaquín González-Ródenas, Ignacio López-Bondia, Rodrigo Aranda-Malavés, Andrés Tudela-Desantes, M. Teresa Anguera

**Affiliations:** ^1^Faculty of Sport Sciences, University of Valencia, Valencia, Spain; ^2^Department of Recreation and Sport Pedagogy, Ohio University, Athens, OH, United States; ^3^Escuela de Doctorado, Universidad Católica de Valencia San Vicente Mártir, Valencia, Spain; ^4^Faculty of Psychology, Institute of Neurosciences, University of Barcelona, Barcelona, Spain

**Keywords:** offensive process, goal scoring opportunities, mixed methods, systematic observation, tactics, performance analysis, soccer

## Abstract

Performance analysis in complex sports like soccer requires the study of the influence of the interaction between both teams during the game on final performance. The mixed methods approach involves the collection, analysis, and interpretation of qualitative and quantitative data for the same purpose and within the framework of the same study. To build certain observation tools, mixed methods are necessary in order to take advantage of integration between qualitative and quantitative elements. The aim of this study was to develop a new no standard observation tool to analyze soccer offensive performance considering not only the observed team but also some aspects of the opponent behavior, as well as to test its reliability. The process consisted in expert meetings and exploratory observations. Experts carried out several design and re-design steps of the observation tool to its final form which includes two macro-criteria and 31 dimensions. The basic unit of analysis was the “team possession” and the main aims of study were: (a) technical, tactical and spatial characteristics of the start, the development and the end of the team possession and its offensive performance, (b) the behavior of the observed team just after losing the ball possession and its defensive performance. Inter-observer and intra-observer analysis were carried out and kappa coefficient was calculated to test the observation tool reliability and improve the quality of data. Results indicate that optimal inter and intra-reliability levels obtained in this work are high enough as for suggesting that the observation tool for offensive performance in soccer (REOFUT) could be an adequate tool for analyzing offensive play actions and their performance in soccer.

## Introduction

Researchers have been mixing qualitative and quantitative approaches since the last 20 years (Tashakkori and Teddlie, 1998; Teddlie and Johnson, 2009), but mixed methods research represents “a new movement, or discourse, or research paradigm (with a growing number of members) that has arisen in response to the currents of qualitative research and quantitative research” (Johnson et al., 2007, p. 113). Many researchers do not mix qualitative and quantitative approaches in optimal ways, according to Powell et al. (2008), even when qualitative techniques can be used to enhance the development of quantitative instruments and vice versa (Collins et al., 2006). The mixed methods potential is very broad, and includes instrument fidelity, “maximizing the appropriateness and/or utility of the instruments used, whether quantitative or qualitative” (Onwuegbuzie et al., 2010, p. 57).

The goal of soccer performance analysis is to provide accurate, clear and objective information to players, coaches and clubs to improve future team performance (McGarry, 2009). In this sense, the development of new technological systems [e.g., Global Positional System (GPS), Prozone-STATS, and OPTA] has contributed to increase the quantitative based knowledge in this sport. Notational analysis, which consists of the technique by which the events that take place during the game are recorded (James, 2006) has provided and continues to provide important information for coaches and players, thus helping to improve the training process (Borrie et al., 2002).

However, there is a need to use a new methodological approach based not only on quantitative methods but also on qualitative ones. Indeed, some authors report a growth and proliferation of new methodological approaches to collect and analyze data (Sarmento et al., 2018a). In soccer, there is a need for methods that add qualitative value to performance analysis in order to increase the validity and possible applications of research. This makes necessary the implementation of a mixed methods approach that involves the collection, analysis, and interpretation of qualitative and quantitative data for the same purpose, and within the framework of the same study, on mixed methods (Anguera et al., 2018).

Soccer is a complex and multifactorial sport where the two opponent teams generate an unexpected and changing context at each moment of the game, having a constant adaptation to the characteristics caused by the confrontation between them (Gréhaigne et al., 1997). A large part of recent literature in soccer notational analysis has focused on studying offensive performance indicators such as starting zone, number of passes, duration or type of attack, and their influence on offensive performance (Hughes and Franks, 2005; Tenga et al., 2010; Wright et al., 2011; Lago-Ballesteros et al., 2012; Sarmento et al., 2018b). However, despite the complex and interdependent nature of the game action in soccer, there are still few studies focused on analyzing the effect of the interaction between offensive factors on performance while considering defensive factors of the opponent team and contextual factors (Mackenzie and Cushion, 2013). These same authors and others underline the need to implement research methodologies that show detailed definitions with the aim of facilitating the understanding of results and comparison with other studies (Mackenzie and Cushion, 2013; Sarmento et al., 2018a).

Therefore, according to reviews of the literature in performance analysis (Mackenzie and Cushion, 2013; Sarmento et al., 2014) the future of game analysis in soccer requires the building of observation instruments that integrate the study of criteria related to the interaction with the opponent. In this type of instruments, the use of qualitative attributes where the interpretation of the events by the observers is necessary for the collection of the data, can increase the ability to describe the actions developed in the game (Hughes and Bartlett, 2002).

Some instruments have been proposed to evaluate individual tactical behaviors. González-Villora et al. (2015) review different instruments for the evaluation of the tactical knowledge of football players. All instruments reported there are used to evaluate the tactical knowledge of the soccer player. We would like to highlight the FUT-SAT (Teoldo et al., 2011), which is conceived to assess the tactical knowledge of the player in game situations, both of training and competition.

However, the difficulty of interpreting the team actions in a complex, dynamic and situational context such as a soccer match makes it necessary to correctly define the criteria to limit the errors of interpretation and codification (James et al., 2007), as well as to assess the validity and reliability of the data collected to be analyzed (Hughes et al., 2004).

Therefore, in order to maximize the appropriateness and/or utility of the instruments used, whether quantitative or qualitative, the aim of this work is to describe the process of building and optimizing the development of a qualitative instrument, which will enable to integrate qualitative records with a quantitative analysis. Thus, this work developed a new observation instrument from a mixed method perspective to analyze the offensive collective performance and the tactical characteristics of the action of collective offensive game in soccer, considering the behavior of the opponent team and also contextual dimensions, it also verified the reliability of such instrument, bearing in mind that this methodology is ideal for research on behaviors that occur in soccer (Anguera and Hernández-Mendo, 2013, 2014).

## Materials and Methods

### Participants

No experimental analysis involving human studies is performed in the study. Six experts were included in this process, who met the following criteria: (1) graduate in physical activity and sport sciences, (2) soccer coach license UEFA A, (3) more than 1 year as a soccer coach in a soccer team of an official competition, (4) postgraduate master in sports sciences or PhD in sports sciences, and (5) experience in performance analysis research (final master’s thesis, doctoral thesis or scientific publication).

The six experts provided written informed consent after details of the study were communicated verbally and in written form prior to participate in the study and it was approved by the Ethics Committee of the University of Barcelona (IRB00003099). In order to carry out the second, third, and fifth stage of the study, in which observation was necessary, a direct, non-participatory, systematic, and natural observational methodology was used (Anguera et al., 2011) (see the “Procedures” section). Matches were recorded from TV broadcasters and were registered and analyzed post-event. Because the video recordings were public, confidentiality was not an issue, and authorization was not required from the players observed or their representatives. Furthermore, the information cannot be considered either personal or intimate, as the research consisted solely of naturalistic observations in public places, and it was not anticipated that the recordings would be used in a manner that could cause personal harm (The American Psychological Association’s [APA’s], 2010).

### Procedures

The design of the research instrument was carried out in five stages:

In the first stage, a literature review on tactical analysis in soccer was carried out in order to know the performance analysis methods used in previous studies. Afterward, the group of experts held an initial meeting where the basic objectives of the instrument were defined, as well as the innovative aspects that were intended to be included. In this sense, it was decided that the main objective of the observation instrument was to analyze the tactical dimensions that could influence the attainment of offensive performance in soccer. Another objective was to study the behavior carried out by a team after losing possession of the ball in play in order to know the tactical behavior in the transition between offensive and the subsequent defensive action. It was decided that the basic unit of study was the “possession” of the team’s ball according to the definition of Pollard and Reep (1997) but also to observe some dimensions of the immediately subsequent possession carried out by the opponent team if the “possession” ends but the game continues. Pollard and Reep (1997) define “possession” as:

*“A team possession starts when a player gains possession of the ball by any means other than from a player of the same team. The player must have enough control over the ball to be able to have a deliberate influence on its subsequent direction. The team possession may continue with a series of passes between players of the same team but ends immediately when one of the following events occurs: a) the ball goes out of play; b) the ball touches a player of the opposing team (e.g., by means of a tackle, an intercepted pass or a shot being saved). A momentary touch that does not significantly change the direction of the ball is excluded, or c) the regulation is violated (out of play or foul)*.”

In the second stage, the design and development of the dimensions and their respective categories were carried out. This stage was characterized by several meetings based on the definition, discussion and re-definition of the dimensions and categories of the instrument separated by individual exploratory observation practices where the experts collected, suggestions or contributions resulting from the application of the preliminary research instrument. All those considerations were discussed in the following meetings in order to re-design the proposed dimensions and categories, or to add new ones that will complete a better analysis. In these observation practices soccer matches of the Spanish national team corresponding to the final phase of the South Africa 2010 FIFA World Cup (Spain-Portugal, Spain-Paraguay and Spain-Holland) were used. The first version of REndimiento Ofensivo en FÙtbol (REOFUT; in English: offensive performance in soccer) ended when each category system met the requirements of completeness and mutual exclusivity (Anguera et al., 2007) and the experts did not consider necessary new dimensions. In this sense, the completeness was considered when any behavior under analysis could be assigned to one of the categories proposed in each dimension while the mutual exclusivity was fulfilled when there was no overlap of the categories and each analyzed behavior was assigned to a single category.

In the third stage, a study was carried out by one of the researchers for the analysis of the offensive game of the Spanish soccer national team in the FIFA World Cup in South Africa 2010, the champion team (852 possessions were analyzed from 7 played matches), using the newly designed first version of REOFUT (González-Ródenas, 2013). The reliability was verified through the analysis of 20 randomly selected possessions of the Spain-Germany match.

In the fourth stage, a series of meetings of the 6 participating experts took place to analyze and discuss the results obtained in the first study in order to verify the usefulness of the criteria and categories of the first REOFUT design. These meetings allowed to eliminate, redesign, redefine criteria, as well as to add other criteria that might be necessary for a better understanding of tactical performance analysis in soccer. As a result of these meetings, the REOFUT was modified from its initial design of 3 macro criteria and 45 dimensions to two macro criteria and 31 dimensions that make up the second version of the instrument. Additionally, several dimensions were redefined as well as categories were grouped or eliminated in order to make the process of data collection and analysis of results more operative and effective.

Finally, once the final design of the instrument was built, the fifth stage was reached where a macro study was carried out in order to analyze the tactical dimensions related to offensive performance, using a sample of Major League Soccer matches. In this macro study, the verification of the reliability of REOFUT took place through the assessment of the agreement between observers (inter-observers) and the analysis of interpretative stability (intra-observer). For the study of the inter-observer agreement, apart from the analysis carried out by the main researcher, a person was trained (Bachelor of Science in Physical Activity and Sport, Master in Research in Sports Science and UEFA A coaching license) during 4 weeks (40 h) in the analysis of the offensive collective game with REOFUT. After the training period, the two observers separately analyzed a game composed of 128 “possession units” of a Major League Soccer professional team in the United States. Regarding the intra-observer agreement, the principal investigator performed the same analysis, 4 weeks after the first analysis, to minimize task familiarity (Robinson and O’Donoghue, 2007), without performing any type of analysis during this time, to check the temporal stability of the analysis performed. To assess the levels of agreement, Kappa correlation coefficients (k) were calculated and analyzed according to the classification by Altman (1991), where the values between 0.8 and 1.0 were considered very good agreement, 0.61–0.80 good, 0.41–0.60 moderate, 0.21–0.40 low and <0.21 very low.

### Observational Instrument

The REOFUT observational instrument is a combination of field format and category systems (Anguera et al., 2007). As it is shown in the conceptual approach, the field format allows the appropriate location of the qualitative dimensions. Based on each of these dimensions, when the criteria of having a theoretical framework and timelessness are fulfilled, a system of qualitative categories has been developed. That obeys the double requirement of completeness and mutual exclusivity. In addition, as suggested by Mackenzie and Cushion (2013) and Sarmento et al. (2014) this instrument includes contextual dimensions such as location of the match (home, away, neutral), momentary result (winning, losing, drawing), team and opponent level (high, medium or low depending on their position in the classification) and time of game (first part, second part, extra time).

**FIGURE 1:**
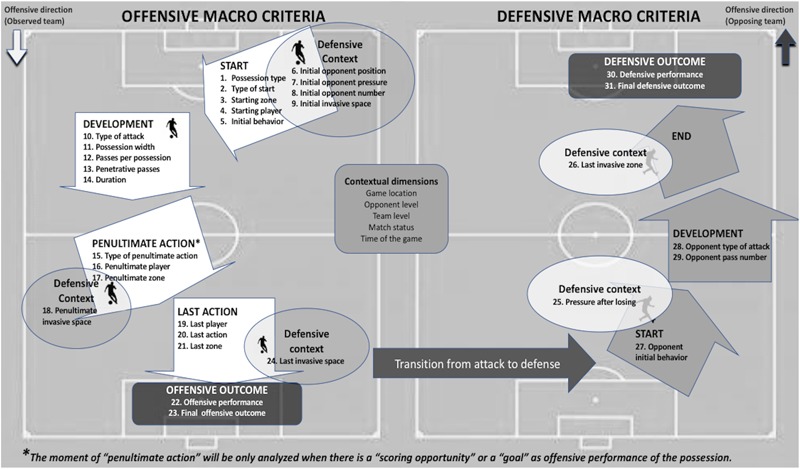
Scheme of the macro-criteria and dimensions that REOFUT record.

As it is shown in Figure 1, the REOFUT analyzes two large macro-criteria, the offensive where the observed team has possession of the ball and the defensive only in case the rival team manages to recover the ball in play and initiate a subsequent attack. In this way the REOFUT allows us to study four out of the five important moments of the game according to the classification of Hewitt et al. (2016), such as the offensive transition, the positional attack, the offensive set pieces and the defensive transition. Within the macro offensive criterion, 5 temporary successive moments from each possession are analyzed: start of the possession (Table 1), development of the possession (Table 2), penultimate action of the possession (Table 3), end of the possession and performance outcome (Table 4) and the following possession (Table 5), with the singularity that the moment relative to “penultimate action” will only be studied in the case of the existence of a goal or goal opportunity, with the aim of deepening the analysis of the tactical factors related to the attainment of offensive success. For the defensive macro-criterion, four temporary successive moments of the game action subsequent to the loss of the ball are analyzed, with dimensions relative to the start (Table 1), development (Table 2), final (Table 4), and performance outcome of the opponent subsequent team’s possession (Table 5).

**Table 1:** Description and categories for the dimensions related to the start of the possession.

POSSESSION START
**Observed team (offensive)**
1-Possession type: way to start a team possession according to if the ball is in play or out of play. Three categories were considered:
A) Recovery: when a player gains the possession of the ball by any means other than from a player of the same team with the ball in play. B) Set-plays: (1) the restart takes place in the opponents’ half, (2) the tactical situation of the attacking team is prepared to try to shot at goal (both teams group players into or just in front of the box and player positions change because some of the defenders move forward to try to shot at goal) and (3) the attacking team try to cross the ball into the box or shot at goal in one or two passes. (All corner kicks, all penalty kicks and those free kicks with the above characteristics are considered in this category). C) Re-starts: the re-start takes place in any part of the field, (2) the tactical situation of the attacking team is not prepared to try to shot at goal in one or two passes (player positions do not change) and (3) the attacking team try to pass the ball and build up a ball possession. (Goal kicks, free kicks, kick off, throw in).
2-Type of start: individual action by which the possession starts, based in previous studies (Gómez et al., 2012; Barreira et al., 2014). Two categories and eleven sub-categories were considered:
A) When the ball is not in play: the ball is out of play and it is restarted by an individual action: A.1 goal kick; A.2 throw in; A.3 free kick; A.4 corner kick; A.5 kick off; A.6 penalty kick; A.7 dropped ball. B) When de ball is in play: the ball is in play and a team possession begins with an individual action: b.1 Turnover won: when the defender collects, somewhere in the pitch, a ball lost (after clearances or missed passes) by the opposing team (Gómez et al., 2012). b.2 Interception: when a player of the team that has not the ball prevents a ball passed by an opponent with the ball from reaching its intended receiver by contacting the ball and keeping his own team in possession of the ball (Barreira et al., 2014). b.3 Steal: when the defender dispossesses the opponent of the ball through a physical challenge or defensive pressure (Barreira et al., 2014). b.4 Possession gained by the goal-keeper: when the goal-keeper of the team collects a turnover, intercepts or steal the ball.
3-Field starting zone: zone on the field of play where the possession starts (Figure 3). Four categories and sixteen sub-categories were considered:
A) Defensive sector: *zones 1, 2, 3 and 4*. B) Pre-defensive sector: *zones 5, 6, 7 and 8*. C) Pre-offensive sector: *zones 9, 10, 11 and 12*. D) Offensive sector: *zones 13a, b; 14a, b, c, d; 15a, b, c, d and 16a, b*.
4-Starting player: specific position of the player who performs the initial action of the possession. Seven categories were considered depending on the system of play used by the team (Figure 4): (A) Goal-keeper, (B) Central defender, (C) Full back, (D) Central defensive-midfielder, (E) Central offensive-midfielder, (F) Winger and (G) Forward.
5-Initial behavior: degree of offensive directness in the first three seconds of the team possession (Figure 5). Four categories were considered:
A) Non-penetrative action: any technical action towards any direction that does not past opponent player (s) performed during the first three seconds of the ball possession. B) Penetrative action: passes or dribbles towards the opponent’s goal past opponent player (s) performed during the first three seconds of the ball possession. C) Long ball: aerial pass towards the opponent’s goal with no clear advantage for the offensive team, forcing a duel between a teammate and an opposing player. D) Other initial behavior: any other behavior different from the above.
**Opponent defensive situation**
6-Initial opponent position: opponent’s height position on the field when the team possession starts (excluding goalkeeper) (Figure 3). Three categories were considered:
A) Low position: the opponent has the most backward player closer to their own goal line than the midline. B) Medium position: the opponent has the most backward player closer to the midline than to their own goal. C) Advanced position: the opponent has the most backward player in the opposing half.
7-Initial opponent pressure: distance between the player with the ball (first attackers) and an immediate pressing opponent player(s) (first defender(s)) during the first three seconds of the ball possession (Tenga et al., 2009; Lago-Ballesteros et al., 2012). Two categories were considered.
A) Pressure: one or several opponent players press the attackers within the first 3 seconds of the possession (the pressing defender(s) are always located within 1.5 meters from the first attackers). B) No pressure: There is not any player that pressures the attackers during the first 3 seconds of the possession.
8- Initial opponent number: number of defending players located between the ball and their goal when the possession starts (excluding goalkeeper). Three categories were considered:
A) Micro-group: 3 or less defending players. B) Meso-group: 4-6 defending players. C) Macro-group: 7 or more defending players.
**Opponent defensive situation**
9-Initial opponent invasive space: area within the space of defensive occupation (SDO) of the opponent where team possession starts (Figure 3). Four categories and ten sub-categories were considered:
A) Non-invasive zone: a.1: CF. B) Medium-invasive zone: b.1: CM, b.2 MR and b.3 ML. C) Very-invasive zone: c.1 CD, c.2 DR and d.3 DL. D) High-invasive zone: d.1: CB, d.2 BR and d.3 BL.

**Table 2:** Description and categories for the dimensions related to the possession development.

POSSESSION DEVELOPMENT
**Observed team (offensive)**
10-Type of attack: degree of offensive directness and elaboration during the offensive process (Bangsbo and Peitersen, 2000; Tenga et al., 2009; Sarmento et al., 2010; Lago-Ballesteros et al., 2012). Three categories and five sub-categories were considered:
A) Organized attack: (a) the possession starts by winning the ball in play or restarting the game; (b) in this type of team possession the opposing team is organized defensively or is able to re-organize its collective defensive system during the possession. a.1 Combinative attack: the progression towards the opponent’s goal has high number of non-penetrative and short passes. The circulation of the ball takes place more in width than in depth (Sarmento et al., 2018b) and the intention of the team is to disorder the opponent using a high number of passes and slow tempo (evaluated qualitatively). a.2 Direct attack: the progression towards the opponent’s goal includes a long pass from the back players or goalkeeper to the forward players (evaluated qualitatively); the circulation of the ball takes place more in depth than in width and the intention of the team is to take the ball directly near the penalty area to have opportunities of finishing by using a reduced number of passes and high tempo. a.3 Fast attack: the progression towards the opponent’s goal is fast, using few passes and high percentage of penetrative and short passes; the circulation of the ball takes place in width and in depth (Sarmento et al., 2018b) and the intention of the team is to disorder the opponent using few passes and high tempo (evaluated qualitatively). B) Counterattack: the possession starts by winning the ball in play; the opponent is not organized defensively and is not allowed to re-organize their collective defensive system during the team possession; the progression towards the goal attempts to utilize a degree of imbalance right from start to the end with high tempo (Tenga et al., 2010); the circulation of the ball takes place more in depth than in width, using a high percentage of penetrative passes. The intention of the team is to exploit the space left by the opponent when they were attacking. C) Very short attack: The possession starts by winning the ball in play or restarting the game; and the duration of the team possession is too short to allow the observer to categorize the type of attack.
11-Possession width: use of the four longitudinal lanes of the field space during the team possession. Four categories were considered:
A) One lane: During the possession, the ball moves through one of the four longitudinal lanes. B) Two lanes: During the possession, the ball moves through two of the four longitudinal lanes. C) Three lanes: During the possession, the ball moves through three of the four longitudinal lanes. D) Four lanes: During the possession, the ball moves through four of the four longitudinal lanes.
12-Passes per possession: number of passes performed by the offensive team during the possession.
13-Number of penetrative passes: number of passes performed by the offensive team during the possession towards the opponent’s goal past opponent player(s).
14-Duration: time (in seconds) from the beginning until the end of the possession.

**Table 3:** Description and categories for the dimensions related to the penultimate action of the possession.

**FIGURE 2:**
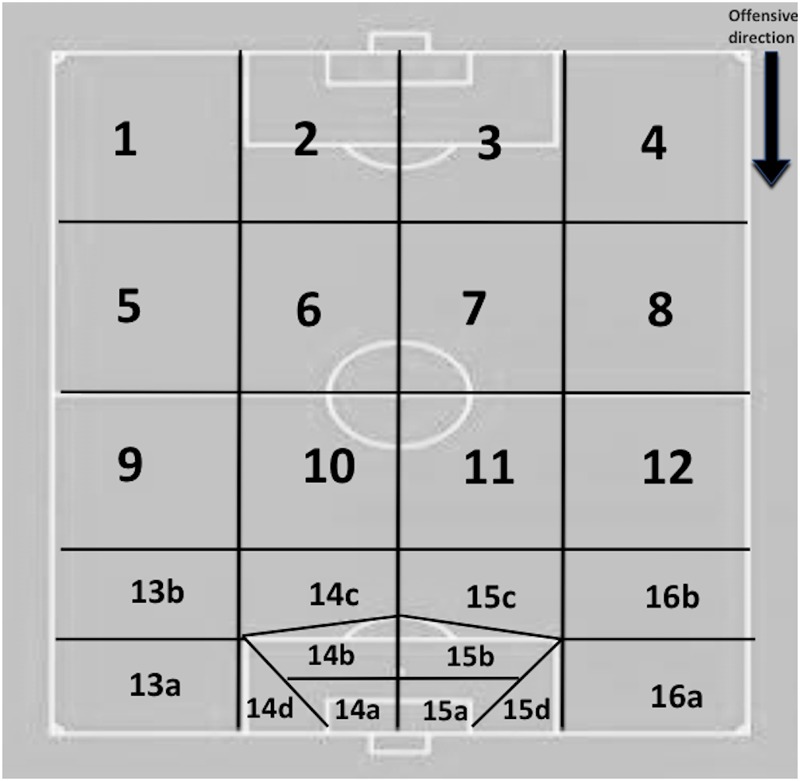
Zones of the field and “score pentagon.” The “score pentagon” is subdivided into different zones in order to perform a more specific analysis of the dimensions related to goals and goal scoring opportunities.

**FIGURE 3:**
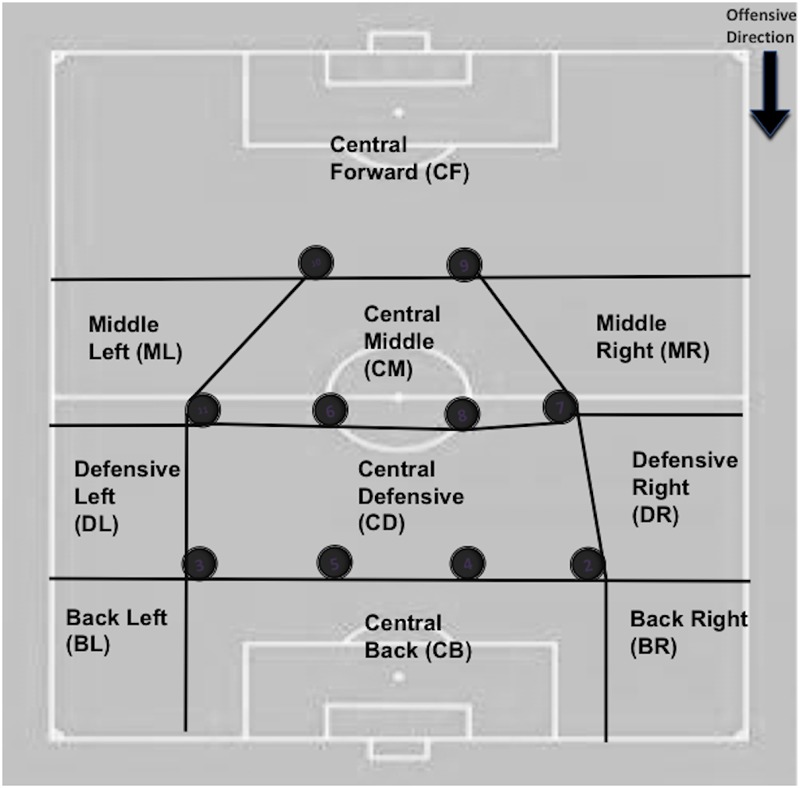
Space of defensive occupation that define the level of invasion over the opponent (Adapted from previous studies, Castellano, 2000; Gréhaigne, 2001; Seabra and Dantas, 2006; Aranda et al., 2009). These zones are dynamic and change every second depending on the positioning on the opposing players.

**FIGURE 4:**
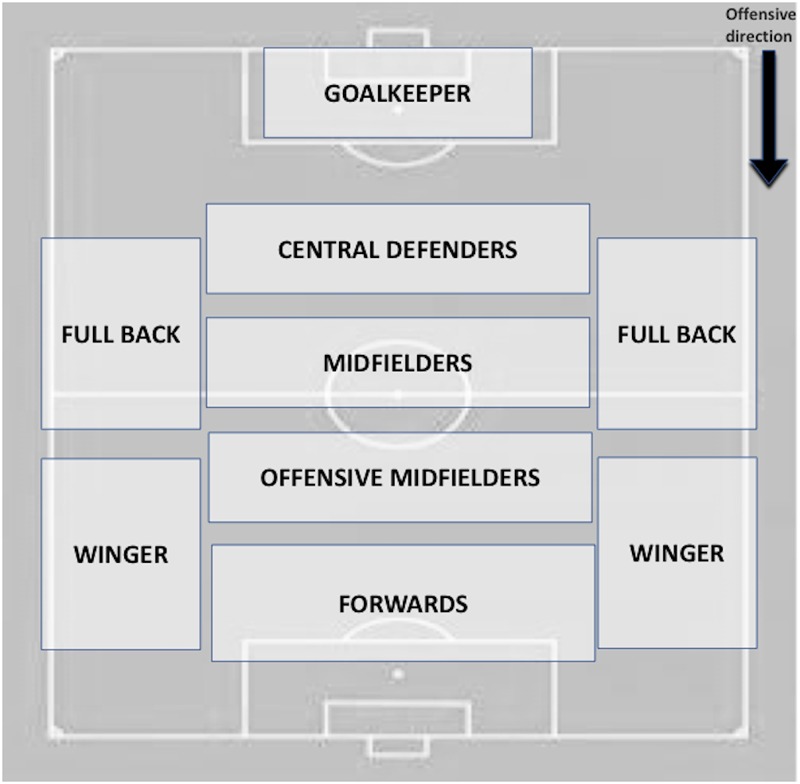
Specific positions within the system of play used by the team in order to determine the player that performs the action. This characterization depends on the system used by each analyzed team.

PENULTIMATE ACTION (only registered if it is followed by a scoring opportunity)
**Observed team (offensive) in the penultimate action**
15-Penultimate action: technical-tactical action performed immediately before the final action that allows the final player to have the opportunity of shooting at goal. This action may be performed by the same player that shoots at goal (individual action) or by a teammate that pass the ball to the final player (collective play). Two categories and seven sub-categories were considered:
A) Individual action: the final player receives the ball without having a scoring opportunity but he achieves to create one by means of an individual action. This category has four sub-categories: a.1 Dribbling: the final player dribbles the ball goal past defenders to create a scoring opportunity. a.2 Running with the ball: the final player carries the ball towards a goal scoring situation. a.3 Collecting a free ball: the final player collects a free ball that allows him to have an immediate scoring opportunity. a.4 Shot from distance: the final player shoots from outside the score pentagon. B) Collective play: The penultimate player in the team possession performs a pass that allows the last player to have an immediate scoring opportunity. This category has three sub-categories. b.1 Pass in behind the defence: pass from central channels of the field that breaks the opposing defensive line and allows the receiver to have an immediate scoring opportunity in front of the goalkeeper. B.2 Cross: pass performed from the wide channels of the field in the opposing half (Figure 1) towards the penalty box (Sarmento et al., 2010) that allows the receiver to have an immediate scoring opportunity. b.3 Goal pass: the final player receives an assist in form of a pass (different from a pass in behind and cross) from a different player that allows him to have an immediate scoring opportunity.
16-Penultimate player: specific position of the player that performs the penultimate action. Seven categories were considered depending on the system of play used by the team (Figure 4): A) Goal-keeper, B) Central defender, C) Full back, D) Central midfielder, E) Central offensive-midfielder, F) Winger and G) Forward.
17-Field Penultimate zone: zone on the field of play where the penultimate action of the possession is performed (Figure 3). Four categories and sixteen sub-categories were considered (Figure 2). Four categories and sixteen sub-categories were considered:
A) Defensive sector: zones 1, 2, 3 and 4. B) Pre-defensive sector: zones 5, 6, 7 and 8. C) Pre-offensive sector: zones 9, 10, 11 and 12. D) Offensive sector: zones 13a, b; 14a, b, c, d; 15a, b, c, d and 16a, b.
**Opponent defensive situation**
18-Penultimate opponent invasive zone: Area within the space of defensive occupation (SDO) of the opponent where penultimate action is done (Figure 3). Four categories and ten sub-categories were considered:
A) Non-invasive zone: a.1: CF. B) Medium-invasive zone: b.1: CM, b.2 MR and b.3 ML. C) Very-invasive zone: c.1 CD, c.2 DR and d.3 DL. D) High-invasive zone: d.1: CB, d.2 BR and d.3 BL.

**Table 4:** Description and categories for the dimensions related to the end of the possession.

END OF POSSESSION
**Observed team (offensive) final action**
19-Last player: it refers to the position that the player that does the last action has into the team system. Eleven categories were considered (Figure 4).
20- Last action: technical-tactical action performed by the last player who played the ball in that possession. It considers the spatial situation of the opponent team at the moment in which the action is done (Figure 2). Twelve categories were considered:
A) Non-penetrative pass: the possession ends after a pass towards any direction that does not past opponent player (s). B) Penetrative pass: the possession ends after an unsuccessful pass towards the opponents goal past opponent player(s). C) Pass in behind the defense: the possession ends after a pass from inside zones of field that try to break the defensive line of the opponent. D) Cross: the possession ends after a pass from the two lateral lanes of the field towards the penalty box in the opponent’s half field (Sarmento et al., 2010). E) Goal pass: the possession ends after any other pass that intended a goal opportunity not categorized into pass in behind the defense nor into cross. F) Long pass: the possession ends after an aerial pass towards the opponent’s goal with no clear advantage for any teammate, before the expected challenge. G) Dribbling: the possession ends with an unsuccessful dribbling towards the opponent’s goal past opponent player (s). H) Shoot with 1 contact: the possession ends with a shot on goal by means of a single contact (goal or not). I) Shoot with 2 or more contacts: the possession ends with a shot on goal by means of two or more contacts (goal or not). J) Header: the possession ends with a head kick on goal. K) Challenge: the possession ends while a challenge after a long pass. L) Another action: the possession ends after any other action not categorized into any of the previous categories.
21-Field last zone: zone on the field of play where the last action of the possession is performed (Figure 3). Four categories and sixteen sub-categories were considered (Figure 2):
A) Defensive sector: zones 1, 2, 3 and 4. B) Pre-defensive sector: zones 5, 6, 7 and 8. C) Pre-offensive sector: zones 9, 10, 11 and 12. D) Offensive sector: zones 13a, b; 14a, b, c, d; 15a, b, c, d and 16a, b.
22-Offensive performance: Degree of offensive success of the possession, based on the degree of penetration over the opposing team and the achievement of scoring opportunities and goals (Figure 3). Four categories were considered:
A) Goal: the possession ends in goal. B) Scoring opportunity: the possession ends with a clear chance of scoring a goal during team possession. This include all the chances of shooting than one player has inside the score pentagon (the player is facing the goal, there is not any opponents between him and the goal and he has enough space and time to make a playing decision). There are also considered scoring opportunities all shoots from outside the score pentagon that pass near the goal (2 meters or less with respect to the goal). C) Offensive penetration: The team possession achieves to beat the forwards and midfielders’ lines of the opponent and face directly the defensive line during the offensive sequence but the possession ends without creating any scoring opportunity. The player(s) facing the defensive line has/have enough time and space to perform intended actions on the ball at the moment of receiving the ball. D) No offensive penetration: The team possession does not achieve to disorder and beat the forwards or midfielders’ lines of the opposing team during the offensive sequence.
23-Final offensive outcome: type of possession that follows to the observed offensive possession considering if the ball is still in play or not, which team has the ball and what type of possession is the following one. Six categories were considered:
A) Set play for: the following possession is a set play for the observed team. B) Set play against: the following possession is a set play for the opponent team. C) Restart for: the following possession is an offensive restart but not set play for the observed team. D) Restart against: the following possession is offensive restart but not set play for the opponent team. E) Neutral ball: The ball is lost in the field (after clearances or missed passes) but neither team possess the ball, so a physical duel is required to gain or regain the ball possession. F) Opponent recovery: the following possession is an offensive recovery of the opponent team (by interception, steal or turnover of the ball).
**Opponent defensive situation in the final action**
24-Last opponent invasive zone: area within the space of defensive occupation (SDO) of the opponent where last action is done (Figure 3). Four categories and ten sub-categories were considered:
A) Non-invasive zone: a.1: CF. B) Medium-invasive zone: b.1: CM, b.2 MR and b.3 ML. C) Very-invasive zone: c.1 CD, c.2 DR and d.3 DL. D) High-invasive zone: d.1: CB, d.2 BR and d.3 BL.

**Table 5:** Description and categories for the defensive dimensions if the following possession is against.

**FIGURE 5:**
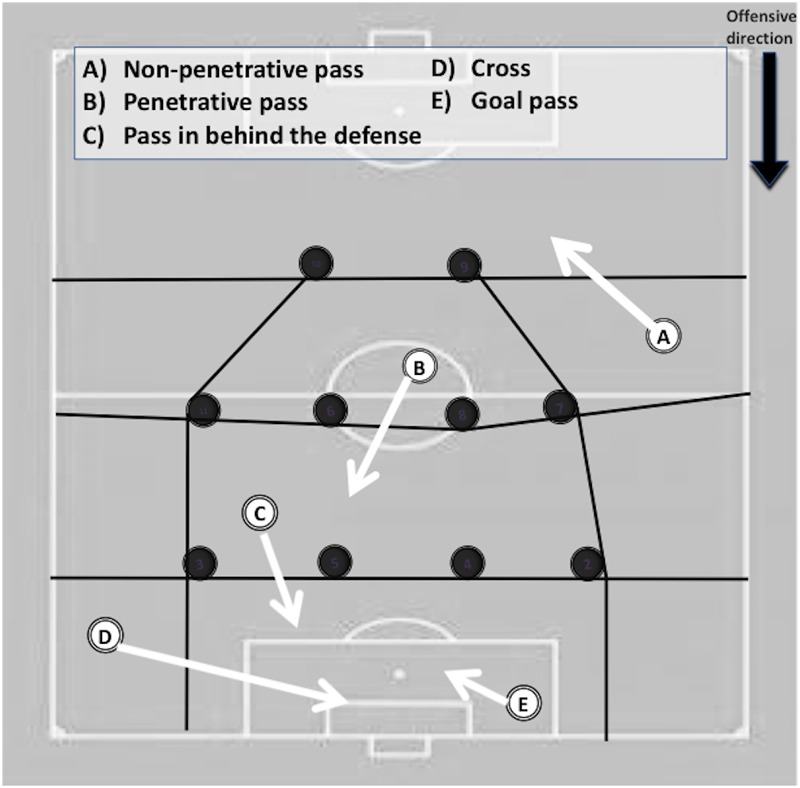
Example of different tactical behaviors related to the penetration over the opponent and their tactical performance.

FOLLOWING OPPONENT POSSESSION START
**Observed team (defensive)**
25-Pressure after losing the ball: Distance between the opponent player with the ball (first attackers) and an immediate pressing player(s) (first defender(s)) during the first three seconds of the ball possession (Tenga et al., 2009; Lago-Ballesteros et al., 2012). Two categories were considered.
A) Pressure: one or several players press the attackers within the first 3 seconds of the possession (the pressing player (s) are always located within 1.5 meters from the first attackers). B) No pressure: any player presses the attackers during the first 3 seconds of the possession.
**Opponent team (offensive)**
26-Opponent initial offensive behavior: degree of offensive directness in the first three seconds of the opponent team possession (Figure 5). Four categories were considered:
A) Non-penetrative action: technical-tactical actions are performed towards any direction that does not past any player (s) during the first three seconds of the ball possession. B) Penetrative action: opponent passes or dribbles towards the goal past player (s) performed during the first three seconds of the ball possession. C) Long ball: aerial pass towards the opponent’s goal with no clear advantage for the offensive team, forcing a duel between a teammate and an opposing player. D) Other initial behavior: any other behavior.
**FOLLOWING OPPONENT POSSESSION DEVELOPMENT**
**Observed team (defensive)**
27-Type of attack: degree of offensive directness and elaboration during the offensive process (Bangsbo and Peitersen, 2000; Tenga et al., 2009; Sarmento et al., 2010; Lago-Ballesteros et al., 2012). Three categories and five sub-categories were considered:
A) Organized attack: (a) the possession starts by winning the ball in play or restarting the game; (b) in this type of team possession the opposing team is organized defensively or is able to re-organize its collective defensive system during the possession. a.1 Combinative attack: the progression towards the opponent’s goal has high number of non-penetrative and short passes. The circulation of the ball takes place more in width than in depth (Sarmento et al., 2018b) and the intention of the team is to disorder the opponent using a high number of passes and slow tempo (evaluated qualitatively). a.2 Direct attack: the progression towards the opponent’s goal includes a long pass from the back players or goalkeeper to the forward players (evaluated qualitatively); the circulation of the ball takes place more in depth than in width and the intention of the team is to take the ball directly near the penalty area to have opportunities of finishing by using a reduced number of passes and high tempo. a.3 Fast attack: the progression towards the opponent’s goal is fast, using few passes and high percentage of penetrative and short passes; the circulation of the ball takes place in width and in depth (Sarmento et al., 2018b) and the intention of the team is to disorder the opponent using few passes and high tempo (evaluated qualitatively). B) Counterattack: the possession starts by winning the ball in play; the opponent is not organized defensively and is not allowed to re-organize their collective defensive system during the team possession; the progression towards the goal attempts to utilize a degree of imbalance right from start to the end with high tempo (Tenga et al., 2010); the circulation of the ball takes place more in depth than in width, using a high percentage of penetrative passes. The intention of the team is to exploit the space left by the opponent when they were attacking. C) Very short attack: The possession starts by winning the ball in play or restarting the game; and the duration of the team possession is too short to allow the observer to categorize the type of attack.
28-Opponent number of passes: number of passes performed by the opposing team during the possession.
**END OF THE FOLLOWING OPPONENT POSSESSION**
**Observed team (defensive) final action**
29-Final observed team invasive zone: Area within the space of defensive occupation (SDO) of the observed team where last action of the opponent team is done (Figure 3). Four categories and ten sub-categories were considered:
A) Non-invasive zone: a.1: CF. B) Medium-invasive zone: b.1: CM, b.2 MR and b.3 ML. C) Very-invasive zone: c.1 CD, c.2 DR and d.3 DL. D) High-invasive zone: d.1: CB, d.2 BR and d.3 BL.
**Opponent team (offensive) final action**
30-Defensive performance: Degree of offensive success of the possession, based on the degree of penetration over the observed team and the achievement of scoring opportunities and goals (Figure 3). Four categories were considered:
A) Goal: the opponent possession ends in goal. B) Scoring opportunity: the opponent possession ends with a clear chance of scoring a goal during team possession. This include all the chances of shoot than one player has inside the score pentagon (the player is facing the goal, there is not any opponents between him and the goal and he has enough space and time to make a playing decision). There are also considered scoring opportunities all shoots from outside the score pentagon that pass near the goal (2 meters or less with respect to the goal). C) Offensive penetration: The opponent possession achieves to beat the forwards and midfielders’ lines of the opponent and face directly the defensive line during the offensive sequence but the possession ends without creating any scoring opportunity. The player(s) facing the defensive line has/have enough time and space to perform intended actions on the ball at the moment of receiving the ball. D) No offensive penetration: The opponent possession does not achieve to disorder and beat the forwards or midfielders’ lines of the opposing team during the offensive sequence.
31-Final defensive outcome: type of possession that follows to the opponent offensive possession considering if the ball is still in play or not, which team has the ball and what type of possession is the following one. Six categories were considered:
A) Set play for: the following possession is a set play for the observed team. B) Set play against: the following possession is a set play for the opponent team. C) Restart for: the following possession is an offensive restart but not set play for the observed team. D) Restart against: the following possession is offensive restart but not set play for the opponent team. E) Neutral ball: The ball is lost in the field (after clearances or missed passes) but neither team possess the ball, so a physical duel is required to gain or regain the ball possession. F) Team recovery: the following possession is an offensive recovery of the team (by interception, steal or turnover of the ball).

The REOFUT contains qualitative spatial localization criteria in the different moments of the game. On the one hand, the formal static space is considered where the game rectangle is subdivided into four transversal sectors (defensive, pre-defensive, pre-offensive, and offensive) and four longitudinal lanes (Mombaerts, 2000). In this formal space, an area called “score pentagon” is delimited (Corberán, 2009), which is the selected space from where there is a distance of less than 20 m from the goal and a high shooting angle, factors that are key for the achievement of the goals (Pollard and Reep, 1997; Ensum et al., 2005). From the completion pentagon, different areas are subdivided into zones 13 (13a and b), 14 (14a, b, c, and d), 15 (15a, b, c, and d) and 16 (16a and b) in order to be able to perform a more specific analysis of the categories of goal and goal scoring opportunities (Figure 2).

On the other hand, the functional and dynamic concept of space of defensive occupation (SDO) of the rival team is used, which we also call “invasive space” (Figure 3). The SDO is defined by Gréhaigne (2001) as the “space that is constituted by the positions of the players located, at a given moment, in the periphery of a team in play, except the goalkeeper” (positions are showed in Figure 4). The interrelated positions draw a polygonal surface defined as the SDO. The location of the player holding the ball in relation to the SDO of the opposing team during the possession observed has been taken into account based on subdivisions made by previous works (Castellano and Hernández Mendo, 2001; Seabra and Dantas, 2006; Aranda et al., 2009) and 10 categories have been defined in the form of subspaces that show the degree of tactical penetration or invasion of the player in possession of the ball into the SDO drawn by the opposing team at a given time, as well as the situation of that player in the inside or outside of the adversary SDO (Figure 5). The location of the player in possession of the ball in relation to the position of the opponents constitutes a fundamental concept in the definition of many criteria included in the REOFUT (Figure 3).

### Recording Instrument

The fifth stage, which was carried out to analyze the tactical dimensions related to offensive performance of Major League matches, was carried out using the recording instrument LINCE (v.1.2.1) (Gabín et al., 2012). Dimensions and categories of REOFUT were coded and the observation of behaviors by the two observers was done using this software.

### Statistical Analysis

To assess the inter- and intra-observer concordance of the REOFUT, kappa index Cohen’s (1960) was calculated using SPSS 21.0 for Windows (SPSS, Chicago, IL, United States).

## Results

Given the objective of this article, oriented to the construction of an observation instrument, the results refer to the quality control of the data, focused on the intra-observer and inter-observer concordance.

Table 6 shows the Kappa values for each of the dimensions of the observation instrument. We can observe how the values for the intra-observer analysis are higher than those obtained in the inter-observers. In this sense, according to the criterion of Altman (1991) the intra-observer analysis shows how 83.9% of the dimensions present very good reliability (0.81–1.0) and 16.1% good (0.61–0.80). On the other hand, the intra-observer analysis shows how 96.8% of the dimensions present a very high reliability (0.81–1.0) and the remaining 3.2% show a good reliability (0.61–0.80).

**Table 6:** Kappa values obtained for the dimensions of the REOFUT observation tool.

Macro criteria	Moment	Dimension	*n*	*K* inter-observers	*K* intra-observer
OFFENSIVE	START	1. Possession type	128	0.972	1.000
		2. Type of start	128	0.891	0.963
		3. Field starting zone	128	0.958	0.947
		4. Initial player	128	0.951	0.990
		5. Initial behavior	107	0.819	0.963
		6. Initial opponent position	107	0.901	0.943
		7. Initial opponent pressure	107	0.815	0.861
		8. Initial opponent number	107	0.897	0.916
		9. Initial opponent invasive zone	107	0.800	0.862
	DEVELOPMENT	10. Type of attack	107	0.776	0.898
		11. Possession width	107	0.935	0.935
		12. Number of passes	107	0.925	0.947
		13. Number of penetrative passes	107	0.779	0.767
		14. Duration	107	0.958	0.958
	END	15. Penultimate action	16	0.765	0.889
		16. Penultimate action	16	0.938	1.000
		17. Penultimate field zone	16	0.963	0.951
		18. Penultimate invasive zone	16	0.774	0.845
		19. Last player	128	0.979	1.000
		20. Last action	128	0.841	0.883
		21. Last field zone	128	0.959	0.979
		22. Offensive performance	128	0.942	0.942
		23. Possession outcome	128	0.940	0.964
		24. Final opponent invasive zone	107	0.813	0.907
DEFENSIVE	START	25. Pressure after losing the ball	45	0.821	0.940
		26. Opponent initial offensive behavior	45	0.841	0.901
	DEVELOPEMENT	27. Opponent type of attack	45	0.839	0.905
		28. Opponent number of passes	45	0.895	0.948
		29. Last invasive zone	45	0.758	0.946
	END	30. Defensive performance	45	0.824	0.893
		31. Defensive outcome	45	0.905	0.937

## Discussion

The central axis of the work has been to present the process of construction of the observation instrument REOFUT, from the qualitative behavior of the soccer team, to the quantitative recording of data, demonstrating that this tool is suitable and consistent for the analysis of offensive possessions in soccer.

First, this study has presented the design process of REOFUT, which aims to provide a greater contextualization of tactical behavior and offensive performance in soccer supported by a broad theoretical framework as claimed by different authors (Mackenzie and Cushion, 2013; Sarmento et al., 2018a). It is remarkable the fact that it incorporates the concept of SDO as well as the position, numerical balance and pressure of the opponent to analyze the interaction between both teams during different phases of the game. In addition, one of the most novel aspects of the REOFUT is to describe in detail the type of technical-tactical actions that can be carried out during the possession, emphasizing its tactical functionality with respect to the opponent rather than its execution, as some recent studies suggested (McLean et al., 2017). Also, the concept of type of progression in attack of the offensive process is used considering previous studies (Tenga et al., 2009; Sarmento et al., 2010; Lago-Ballesteros et al., 2012) and as main novelty, the offensive performance dimension has been defined by four categories that reflect the degree of offensive penetration achieved by the team observed on the opponent during possession (no depth, deep attack, goal scoring opportunity and goal). Therefore, the REOFUT provides a methodological framework of analysis that allows future researchers the possibility of studying globally or specifically different dimensions of the game, such as possessions that originate goals or goals, defensive transitions, as well as the study of game patterns at different times of possession.

The first step is to correctly record and code the data, and this is where the *ad hoc* observation instrument (REOFUT) is used. The record can be managed and processed systematically within an empirical research setting that ensures replicability. The recorded data can be transformed into a series of complete or incomplete code matrices containing purely qualitative information (Anguera et al., 2017). This transformation is achieved by organizing the dimensions into columns and adding the behavioral units (possessions) to the corresponding rows, ready for quantitative analysis.

Once the necessary data controls (as Kappa coefficient for inter-observers agreement) has been performed, the researcher now has access to a series of code matrices perfectly suited for analysis using different techniques.

Secondly, quality control of the data was carried out through the analysis of agreement according to the proposal of the Kappa index, considered as an appropriate analytical technique to analyze the measure of agreement for categorical data in the analysis of sports performance (Robinson and O’Donoghue, 2007). In this way, it has been observed how the REOFUT dimensions show very good and good levels of reliability in all the dimensions, according to the criteria of Altman (1991). Intra-observer analysis shows better levels of reliability than inter-observers. This fact may be due to the fact that some of the behaviors analyzed require the interpretation by the observers, which may cause discrepancies in the observations in some cases. In this line, James et al. (2007) and Losada and Manolov (2015) argue that the disparity between two observers can be expected especially when the analytical procedure requires considerable skill and experience. In addition, these authors add that factors such as training in the analytical procedure, operational definitions and the nature of the dimensions should be considered in the interpretation of the results. In the present study, high reliability values were obtained with a training period of the observer of 4 weeks and the operational definitions of the analytical procedure were specially specified and studied both theoretically and practically during the exploratory observation phase of the study.

In terms of the nature of dimensions and categories, some observations may naturally be more difficult to perform without errors than others (James et al., 2007). This study has observed how the behaviors in which the interpretation by the observers was more relevant have obtained lower agreement values than the behaviors with lower interpretative demands. For example, the dimensions referred to the location of the initial action, penultimate and last action in the rival invasive space obtained lower values of agreement than the dimensions related to the location of them in the formal game space, both inter-observers (0.800, 0.776, 0.813 vs. 0.958, 0.963, 0.959) as intra-observer (0.862, 0.845, 0.907 vs. 0.947, 0.951, 0.979), respectively. This result must be understood in relation to the fact that the zones of the opponent SDO are changing with the evolution of the game action, which requires more experience for interpretation by the observers, while the formal field zones are invariable and the references on the field help to draw imaginary lines on the playing field that facilitate location.

In relation to similar studies, our results coincide with previous investigations that found how the spatial categorization considering the SDO of the equipment can be a reliable measure for the analysis of the game space in soccer. For example, Seabra and Dantas (2006) divided the SDO into 7 sub-spaces to analyze the finalization space in soccer and showed values between 0.83 and 0.93 for the intra-observer analysis and between 0.73 and 0.90 for the inter-observers, which approximates the values found in the present study. Also, Castellano et al. (2000) created an observational tool to analyze actions and contexts of interaction in soccer considering the actions of the players and their relationship with the positioning of the team itself and rivals, showing satisfactory levels of inter-observer and intra-observer reliability.

On the other hand, the present study presents higher concordance values in comparison with the study carried out by Tenga et al. (2009), who developed an observation instrument for soccer performance analysis with 22 dimensions. It is worth noting that the defensive dimensions presented by Tenga et al. (2009) based on pressure, coverage and positioning of the defenders obtained a very low reliability. In addition, as it occurs with REOFUT, this study showed greater concordance for the intra-observer analysis (73% of the criteria very good, 23% good and 4% moderate) than for the inter-observers (32% very good, 23% good, 32% moderate, 9% reasonable, and 4% poor).

In addition, our study presents lower reliability values with respect to the instrument designed by Sarmento et al. (2010) for the analysis of the offensive process in soccer, which achieved reliability values above 0.95 in all the dimensions studied. This instrument aims to detect temporary patterns at the start, development and end of the offensive sequences and shares with the REOFUT the importance of the dimension related to the type of attack, which was divided into categories such as rapid attack, positional attack and counterattack.

REOFUT shows several limitations. On the one hand, as it is a tool based on notational analysis and therefore on the observation, interpretation and recording of events that take place during the game. It may not reflect the total complexity and criteria that the offensive play actions represent. On the other hand, this instrument is fundamentally based on the study of the offensive moment. Although it consists of numerous criteria to describe the different moments of the action of open offensive play, which consists of different moments such as the beginning, the development, the penultimate action and the end, it is not considered an instrument of ideal observation for the study of offensive set pieces, which are actions that have different technical and tactical characteristics to open game situations. It would be fantastic to have a single and simple tool to analyze all types of action that are in a game, but given the diversity of actions that occur in football, to analyze the tactical behavior of the team with a single instrument appears almost impossible.

Finally, this instrument has important practical applications. Firstly, the REOFUT tool can be used for researchers to analyze, describe, predict and compare collective offensive performance. Secondly, soccer coaches and match analysts of all levels can use the theoretical framework of REOFUT to evaluate and register the offensive performance of their teams throughout the season, analyzing the tactical progress and using this information for adjusting and improving the coaching process.

In conclusion, the optimal inter and intra-reliability levels obtained in this work are high enough as for suggesting that REOFUT could be an adequate tool for analyzing offensive play actions and their performance in soccer.

## Author Contributions

RA developed the project, supervised the design of the study, and drafted the manuscript. JG-R was responsible for the review of the literature and the drafting of the manuscript, codified the data, and contributed to the analysis and the method section. IL-B collected and codified the data, and performed the analysis. RA-M was responsible for the analysis, data collection, and data handling. AT-D codified the data. MA revised the content critically. All authors approved the submitted version of the manuscript.

## Conflict of Interest Statement

The authors declare that the research was conducted in the absence of any commercial or financial relationships that could be construed as a potential conflict of interest.
